# Phase 1b/2 study of orally administered pexidartinib in combination with radiation therapy and temozolomide in patients with newly diagnosed glioblastoma

**DOI:** 10.1093/noajnl/vdae202

**Published:** 2024-11-22

**Authors:** Joe S Mendez, Adam L Cohen, Midori Eckenstein, Randy L Jensen, Lindsay M Burt, Karen L Salzman, Marc Chamberlain, Henry H Hsu, Marguerite Hutchinson, Fabio Iwamoto, Keith L Ligon, Maciej M Mrugala, Michael Pelayo, Scott R Plotkin, Vinay K Puduvalli, Jeffrey Raizer, David A Reardon, Michael Sterba, Tobias Walbert, Brian L West, Eric T Wong, Chao Zhang, Howard Colman

**Affiliations:** Huntsman Cancer Institute, Salt Lake City, UT, USA; Department of Neurosurgery, University of Utah, Salt Lake City, UT, USA; Oncology Division, Inova Schar Cancer Institute, Fairfax, VA, USA; Department of Neurology, University of Utah, Salt Lake City, UT, USA; Huntsman Cancer Institute, Salt Lake City, UT, USA; Department of Neurosurgery, University of Utah, Salt Lake City, UT, USA; Department of Radiation Oncology, University of Utah, Salt Lake City, UT, USA; Huntsman Cancer Institute, Salt Lake City, UT, USA; Department of Radiology, University of Utah, Salt Lake City, UT, USA; Lantern Pharma, Dallas, TX, USA; Allysta Pharmaceuticals Inc., Bellevue, WA, USA; STORM Therapeutics Ltd., Cambridge, UK; Division of Neuro-Oncology, Department of Neurology, Columbia University Medical Center, New York, NY, USA; Department of Pathology, Dana Farber Cancer Institute and Brigham and Women’s Hospital, Boston, MA, USA; Division of Medical Oncology, Department of Neurology, Mayo Clinic and Mayo Clinic Cancer Center, Phoenix, AZ, USA; Structure Therapeutics, San Francisco, CA, USA; Department of Neurology and Cancer Center, Massachusetts General Hospital, Boston, MA, USA; Department of Neuro-Oncology, The University of Texas MD Anderson Cancer Center, Houston, TX, USA; Clinical Sciences, Oncology, Takeda Pharmaceutical Company Limited, Cambridge, MA, USA; Center for Neuro-Oncology, Dana-Farber Cancer Institute, Boston, MA, USA; Orbus Therapeutics Inc., Palo Alto, CA, USA; Department of Neurology and Neurosurgery, Henry Ford Health, Wayne State University and Michigan State University, Detroit, MI, USA; Cytoscient LLC, Berkeley, CA, USA; Division of Hematology/Oncology, Rhode Island Hospital, Providence, RI, USA; Tupos Therapeutics Inc., Hayward, CA, USA; Huntsman Cancer Institute, Salt Lake City, UT, USA; Department of Neurosurgery, University of Utah, Salt Lake City, UT, USA

**Keywords:** CSF1R, glioblastoma, KIT, tumor microenvironment, pexidartinib

## Abstract

**Background:**

Glioblastoma (GBM) has a median survival of <2 years. Pexidartinib (PLX3397) is a small-molecule inhibitor of CSF1R, KIT, and oncogenic FTL3, which are implicated in GBM treatment resistance. Results from glioma models indicate that combining radiation therapy (RT) and pexidartinib reduces radiation resistance. We added pexidartinib to standard-of-care RT/temozolomide (TMZ) in patients with newly diagnosed GBM to assess the therapeutic benefit of altering the tumor microenvironment with pexidartinib.

**Methods:**

In this open-label, dose-escalation, multicenter, Phase 1b/2 trial, pexidartinib was administered in combination with RT/TMZ followed by adjuvant pexidartinib + TMZ. During Phase 1b, pexidartinib was given 5 or 7 days/week at multiple dosing levels. The primary Phase 1b endpoint was the recommended Phase 2 dose (RP2D). Phase 2 patients received the RP2D with the primary endpoint of median progression-free survival (mPFS). Secondary objectives were median overall survival (mOS), pharmacokinetics, and safety.

**Results:**

The RP2D of pexidartinib was 800 mg/day for 5 days/week during RT/TMZ, followed by 800 mg/day for 7 days/week with adjuvant TMZ. mPFS was 6.7 months (90% CI: 4.5, 11.5) for the modified intention-to-treat population. The actual mOS was 13.1 months (90% CI: 11.5, 24.5), and the mOS corrected for comparison with matched historical controls was 18.8 months (95% CI: 12.6, 28.0).

**Conclusions:**

This trial established the RP2D of pexidartinib in combination with RT/TMZ and adjuvant TMZ. Pexidartinib was generally safe and well tolerated. Although the study regimen with pexidartinib was not efficacious, pharmacodynamic studies showed modulation of systemic markers that could lead to alteration of the tumor microenvironment.

**Key Points**

- The recommended Phase 2 dose of pexidartinib was 800 mg/day for 5 days/week with radiation therapy (RT)/temozolomide (TMZ) and 800 mg/day for 7 days/week with adjuvant TMZ.
- Pexidartinib combined with RT/TMZ and adjuvant TMZ did not improve median progression-free survival or median overall survival in glioblastoma.
- Pexidartinib induces systemic alterations that may impact the tumor microenvironment.

**Importance of the Study**

Glioblastoma has a dismal prognosis with no significant improvements in outcomes in decades despite clinical trials exploring novel treatment approaches. We explored the potential therapeutic benefit of altering the tumor microenvironment with pexidartinib, a small-molecule inhibitor, in combination with standard-of-care treatment for newly diagnosed glioblastoma. Pexidartinib altered systemic markers that could lead to the alteration of the tumor microenvironment but did not improve progression-free or overall survival. The use of identical eligibility criteria and treatment scheduling parameters relative to defined historical controls in recent Phase 3 trials, as employed in this study, may overcome some limitations associated with a single-arm, nonrandomized Phase 2 design.

Glioblastoma (GBM) is the most common primary malignant brain tumor in adults^1^ and remains one of the most treatment-resistant solid tumors. Radiation therapy (RT) with concurrent temozolomide (TMZ) followed by adjuvant TMZ currently serves as the standard-of-care treatment for newly diagnosed GBM.^2^ However, median survival with standard therapy in modern clinical trials remains <2 years,^3,4^ and the general failure of targeted immunotherapy and antibody-based therapies to improve survival in GBM highlights the need for further advances in treatment.

The tumor microenvironment (TME) plays a key role in the pathogenesis of GBM and is a potential target for novel therapies. Myeloid lineage, bone marrow–derived macrophages, and microglia represent the predominant immune cell population in gliomas.^5^ Evidence suggests that these cell populations play an important role in GBM biology, including promoting tumor progression and treatment resistance. In particular, microglial markers are found in mesenchymal subtypes of GBM that have inferior survival,^6,7^ and the presence of infiltrating microglia correlates with inferior survival.^8^ In addition, a polymorphism (V249I) in the chemokine receptor CX3CR1 is associated with reductions in microglial infiltration and increased survival in GBM patients.^9^ The low percentage of infiltrating T cells in GBM highlights the challenges for T-cell-based immunotherapy approaches in this tumor type, and the general failure to date of checkpoint inhibitors and T-cell-targeted therapies in GBM confirms that T-cell-only immunotherapies are unlikely to be effective. Multiple studies indicate that T-cell entry and activation may be negatively regulated by microglia and macrophages in GBM.^9,10^ These observations suggest that targeting microglia and macrophages to modulate the GBM immune microenvironment, alone or in combination with T-cell-directed therapies, may be a more effective approach to immuno-oncology therapy in GBM.^5^

The feasibility and potential for targeting the microglia- and macrophage-regulated microenvironment as a therapeutic approach in GBM has been demonstrated at both the preclinical and human trial levels. Regulation of key components of the immune microenvironment including microglia, macrophages, osteoclasts, and mast cells is achieved via Fms (or CSF1R), the receptor for colony-stimulating factor 1 (CSF-1, also known as macrophage colony-stimulating factor), and KIT, the receptor for stem cell factor (SCF). High levels of ligands for these regulators, CSF-1 and SCF, have been found in patient-derived glioma cell lines.^11,12^ Microglia depletion in orthotopic GBM models through inhibition of CSF-1 has been shown to decrease spread and tumor burden.^12,13^ Pexidartinib (PLX3397) is an oral, selective small-molecule inhibitor of CSF1R, KIT, and oncogenic FLT3 (the receptor for FLT3 ligand), making it an ideal candidate for a therapeutic approach aimed at altering the TME. Furthermore, pexidartinib has been evaluated in a prior Phase 2, single-agent clinical trial in recurrent GBM and was found to readily cross the blood–brain barrier and be safe in humans.^14^

The proposed use of pexidartinib in newly diagnosed GBM is based on synergism of its impact on the TME and its potential role as a radiosensitizer. Multiple studies have indicated that macrophage/microglia content or state is associated with treatment resistance or increased risk of recurrence.^12,15–17^ In a prostate cancer model, resistance to irradiation was associated with higher levels of tumor-associated macrophages and myeloid-derived suppressor cells, and treatment with pexidartinib resulted in increased radiation sensitivity in this model.^18^ Furthermore, preclinical activity of pexidartinib has been shown to increase radiation sensitivity in distinct GBM models^13,19^ and to demonstrate single-agent activity.^20,21^ Another CSF1R inhibitor, BLZ945, has also shown similar activity in GBM models.^12^

In this study, we sought to explore the potential therapeutic benefit of pexidartinib, administered in combination with standard-of-care RT and concurrent and adjuvant TMZ, based on its influence on the TME and as a potential radiosensitizer in newly diagnosed patients with GBM. In an effort to reduce biases and increase power to reach reasonable efficacy conclusions, the inclusion/exclusion criteria of the current study were chosen to closely match those used in two large and well-powered Phase 3 trials in newly diagnosed GBM (RTOG 0525 and RTOG 0825). This approach ensured that the patient population enrolled in this study closely matched the control groups accrued in those larger trials, facilitating a more equitable comparison with historical controls. Detailed pharmacokinetic (PK) and pharmacodynamic (PD) endpoints were defined to ensure confidence in the concentration and biologic activity of the drug in this population. The specified PK measurements were defined based on a prior Phase 2 presurgical study^14^ to ensure that circulating drug levels and expected brain concentrations were reached. PD biomarkers from blood including circulating plasma CSF-1 and CD14^dim^CD16^+^ mononuclear cell counts, both responsive to pharmacologic inhibition of CSF1R kinase, were evaluated.

## Materials and Methods

### Study Design

This was a prospective, open-label, dose-escalation, multicenter, Phase 1b/2 study of orally administered pexidartinib in combination with RT and TMZ in patients with newly diagnosed GBM; it was designed to evaluate the safety, tolerability, and efficacy of pexidartinib. Nine high-volume brain tumor centers from different regions of the United States took part and enrolled patients in this study (Supplementary Table 1). Approval for this study was obtained from each site’s institutional board review, and informed consent was obtained from all patients prior to participation in the trial. The primary objective of Phase 1b was the identification of the recommended Phase 2 dose (RP2D). For Phase 2, the primary objective was the determination of the median progression-free survival (mPFS). Secondary objectives were the evaluation of median overall survival (mOS) compared with historical controls, PK, safety, and the exploratory endpoint of PD effects of pexidartinib.

The results from our study were compared with those from historical controls from clinical trials RTOG 0525 and RTOG 0825.^3,4^ The inclusion and exclusion criteria of this protocol were specifically written to match those of these prior Phase 3 studies to ensure those studies provided reasonable historical controls for statistical analysis of this study. In addition, the start date of treatment (first day of radiation) was corrected to align day one of treatment in the current protocol with those of the RTOG 0525 and RTOG 0825 studies to increase the accuracy of the comparison of mPFS and mOS relative to each of those trials.

### Patient Selection

Eligible patients were 18 years or older with newly diagnosed GBM (by 2016 WHO definition) or gliosarcoma that was histologically confirmed by partial or gross total resection, with at least 20 unstained slides available. Patients had Karnofsky performance status (KPS) scores of ≥70 and adequate hematologic, hepatic, and renal function by laboratory testing. The study protocol required that RT was received at the participating institution. Exclusion criteria included stereotactic biopsy only, recurrent GBM or metastases, use of carmustine wafers, and/or prior treatment with RT, chemotherapy, or other intratumoral or intracavitary treatment.

### Treatment

In the Phase 1b portion, all patients received a priming dose of 1 week of pexidartinib monotherapy given either continuously (twice daily for 7 days/week) or intermittently (twice daily for 5 days/week Mondays through Fridays [M–F]) with Day 1 considered the first day of the cycle (C1D1). Following this (C1D8), they received standard-of-care combination RT/TMZ with concurrent pexidartinib given either twice daily for 7 days/week or twice daily for 5 days/week M–F. Subsequently, after a 4-week rest period without treatment, all patients received adjuvant TMZ at standard doses (Days 1–5 of a 28-day cycle) with the addition of pexidartinib twice daily (Days 1–28 of the same cycle) for as long as there was no disease progression or unacceptable toxicity. Two pexidartinib dose levels (800 and 1000 mg) were initially planned, with one or more lower doses to be evaluated if the starting dose of 800 mg/day was above the maximum tolerated dose. TMZ was given up to 12 adjuvant cycles whereas there was no limit on the number of cycles of adjuvant pexidartinib (Supplementary Figure 1).

In the Phase 2 portion, patients were treated with the same schedule as outlined above but with concurrent and adjuvant pexidartinib given twice daily for 5 days/week (intermittent dosing) at the RP2D level determined in Phase 1b. In both phases, RT consisted of 2 Gy given once daily 5 days/week (M–F) (total RT dose of 60 Gy using either conformal or intensity-modulated radiation treatment planning); oral TMZ (75 mg/m^2^/day) was administered once daily 7 days/week. After the first cycle of adjuvant therapy, TMZ was escalated to 200 mg/m^2^/day depending on tolerability. After discontinuation of the study drug, patients were to be monitored for OS every 6 months.

### Response Assessment

Patients were monitored for response or disease progression with MRI scans every 2 cycles (8 weeks), with an initial scan obtained 3–4 weeks after completion of RT serving as the baseline scan for determination of response assessment per Response Assessment in Neuro-Oncology criteria.^14^

### Pharmacokinetics and Pharmacodynamic Assessments

#### PK Measurements—

Plasma samples were analyzed for pexidartinib using a validated method (high-performance liquid chromatography [HPLC] or ultra-high-performance liquid chromatography (HPLC) with tandem quadruple mass spectrometric detection) of appropriate specificity and sensitivity in compliance with Good Laboratory Practice standards at WIL Research Laboratories, LLC.

Patient blood samples (4 mL) were collected on Day 1 and on Days 8 (D8) and 15 (D15) at predose and 1, 2, 4, and 6 h after the first dose (all before the second dose). Predose PK samples were obtained on the first day of each cycle after Cycle 1 (C1). Blood samples for PK analysis were collected within a ±15-min window of the scheduled time.

Descriptive statistics were calculated where appropriate for all determined PK parameters. The number of patients (*N*), mean, standard deviation (SD), minimum, median, maximum, geometric mean, and coefficient of variance (CV%) of the geometric mean were calculated per dose cohort. The plasma concentrations for pexidartinib were listed individually and summarized by dose cohort at each time point using descriptive statistics (*N*, mean, SD, minimum, median, maximum, geometric mean, and CV% of the geometric mean).

#### PD Measurements—

Blood samples were collected to evaluate CD14/16 mononuclear cell counts and CSF-1 levels, which are biomarkers of kinase inhibition and myeloid cell recruitment.

For the assessment of CD14/16 mononuclear cell counts, blood samples were collected at baseline and approximately 1 week (5–8 days) after the start of treatment with pexidartinib. Six milliliters of whole blood were collected in a sodium heparin tube, gently inverted, and then stored at 4 °C. Blood samples were shipped to Miraca Life Sciences on the same day as collection. Blood samples were processed and analyzed by flow cytometry on the day of receipt or the day after receipt using the markers listed in Supplementary Table 2. Data files (fcs) were transferred to Plexxikon and analyzed using FlowJo software (FlowJo LLC). The target cell population was quantified according to the procedure in Supplementary Table 3.

For the assessment of CSF-1 levels, blood samples were collected on predose C1D1, C1D8, C2D1, and C3D1. CSF-1 levels were quantified by the Quantikine ELISA (Human M-CSF Immunoassay; R&D Systems, Inc).

Surgical tissue obtained pretreatment was evaluated for O(6)-methylguanine-DNA methyltransferase (MGMT) methylation, CSF-1, CSF1R, tumor-associated macrophages (CD163), and other prognostic biomarkers. More specifically, this presurgical tissue was evaluated by immunohistochemistry for both CSF1 and CD163.

### Statistical Analysis

#### Phase 1b—

The objective of the Phase 1b portion of this study was to determine the RP2D of pexidartinib when combined with concurrent RT and TMZ. Two dose levels were initially planned, with one or more lower doses to be evaluated if the starting dose of 800 mg/day was above the maximum tolerated dose. Seven patients were planned to be accrued at each dose level to ensure that at least 6 patients would be eligible for evaluation of toxicity. A dose level for pexidartinib was considered acceptable if no more than 2 patients of the 6 eligible patients experienced dose-limiting toxicity (DLT). Where all 7 patients were analyzable for adverse events (AEs), only the first 6 were considered in the determination of DLTs. The DLT window was an 11-week period consisting of 1 week of pexidartinib priming, 6 weeks of pexidartinib combined with concurrent RT and TMZ, and the 4-week post-RT recovery period.

#### Phase 2—

For the Phase 2 portion, the primary objective was the determination of mPFS. Secondary objectives included the evaluation of OS, PK, correlative imaging studies, safety, and the exploratory endpoint of PD effects of pexidartinib. The mPFS and mOS were compared with those obtained from the historical controls. PFS and OS were measured from C1D1 using Kaplan–Meier methods. The modified intention-to-treat (mITT) population comprised all patients who received at least 1 dose of the study drug and had no follow-up data. Efficacy analyses were performed primarily on the RP2D population. The RP2D population included all patients in the mITT population who received the RP2D of pexidartinib in either the Phase 1b or Phase 2 portions of the study. The per-protocol population, which consisted of patients who fulfilled the inclusion/exclusion criteria and received a complete 7-week course of combined pexidartinib/RT/TMZ therapy at the RP2D level, was also used for efficacy analysis. The nonparametric Kaplan–Meier method was used to estimate the mPFS, mOS, and median duration of response (DOR). Additional efficacy analyses (PFS, OS, and sensitivity analyses) using a parametric model were based on model parameters estimated by maximum likelihood with a Newton–Raphson algorithm (an exponential distribution of survival times). Specific populations from this study with varying start times of PFS calculation were used to estimate PFS that reflected the corresponding methodologies used to define PFS in the historical controls from the RTOG 0525 and 0825 studies (Supplementary Table 4). The mPFS and its 95% CIs are presented in conjunction with counts of the total number of subjects and number of observed disease progression events. The alternative hypothesis (PFS from historical control) and 1-sided *P*-value based on a 1-sample log-rank test adapted from Finkelstein et al. ^22^ assuming an intercept-only exponential survival model under the null hypothesis, are also presented. The hazard ratio, or estimated hazard rate relative to the hazard rate corresponding to the null hypothesis, is also displayed.

For PFS, estimates are presented with censoring at the earlier date of either the last evaluable tumor assessment or the start of confounding therapy.

As sensitivity analyses, estimates are presented without censoring for confounding therapy and without censoring for confounding therapy or at the last tumor assessment.

For the secondary efficacy analysis, estimates of mOS and its 95% CIs were compared with alternative hypotheses based on historical controls, and *P*-values and HRs are presented as described for PFS (Supplementary Table 4).

For OS, estimates are presented with censoring for confounding therapy, and as sensitivity analyses, estimates are presented without censoring for confounding therapy but with censoring at the last known alive date.

OS was also estimated at 9 and 12 months of follow-up, without censoring for confounding therapy. The expected number of deaths was calculated under an assumed hazard rate of death for the relevant historical control, estimated from their respective mOS, assuming an exponential survival model (Supplementary Table 4). A 1-sided *P-*value, based on the 1-sample log-rank test outlined in Finkelstein et al.^22^ is presented.

In the event that no disease progression or death was documented prior to study termination, analysis cutoff, or the start of confounding anticancer therapy, patients were to be censored for PFS and DOR at the date of the last evaluable tumor assessment.

#### Sample Size and Power—

For the primary endpoint of mPFS, based on a 1-sided log-rank test with a significance level of 0.1 and power of 80%, 22 events (death or progression) in approximately 31 patients were required to detect a 50% relative hazard reduction in PFS due to the addition of pexidartinib compared with the recent historical control mPFS of 5.5 months (RTOG 0525). Assuming ~30% rate of nonevaluability, approximately 37 patients were planned to be enrolled in the Phase 2 portion of the study, which would yield approximately 44 patients when combined with the 7 patients treated at the RP2D in the Phase 1b portion of the study. The primary population for efficacy and safety consisted of the prespecified mITT population, that is, patients who received at least 1 dose of the study drug and had any follow-up data.

## Results

### Patient Characteristics

A total of 65 patients were enrolled across multiple institutions from July 2013 to November 2017. Twenty-two patients were enrolled in Phase 1b and 43 patients in Phase 2 (Supplementary Figure 2). The baseline characteristics of patients are shown in Table 1. The median age of participants was 56 years (range 23–74), and a majority of patients had a KPS of 90–100 (68%). Forty-five percent of patients had a complete resection. Of the 60 patients in whom the tumor MGMT promoter methylation status was known, 30 were methylated and 30 unmethylated. In the overall cohort, 64 of 65 patients stopped participation in the study due to disease progression (*n* = 32), AE (*n* = 16), subject decision/voluntary withdrawal (*n* = 7), other (*n* = 4), investigator decision (*n* = 2), noncompliance (*n* = 2), or death (n = 1). One patient enrolled in the Phase 2 part (Patient 020_011) remains alive and has continued to receive pexidartinib through an expanded access program after this trial was closed. Data related to study medication administered after October 10, 2017, or study visit information after September 12, 2017, for this patient are not included in this report, but survival information has been updated since that date.

**Table 1.** Patient characteristics of patients in Phase 2 (≥15% occurrence in all patients in Phase 2), the combined RP2D groups in Phase 1b and Phase 2 (mITT RP2D population), and the study overall (mITT population)

Characteristic	Combination therapy dose/adjuvant therapy dose^a^		Phase 2 total *N* = 43	Combined RP2D^b^ Phases 1b and 2 *N* = 53	Study overall *N* = 65
	800 mg/800 mg *N* = 27	800 mg/none *N* = 16			
	5 days/7 days	5 days/none		5 days/varies	
Sex					
Male	19 (70)	9 (56)	28 (65)	33 (62)	41 (63)
Female	8 (30)	7 (44)	15 (35)	20 (38)	24 (37)
Age (years)					
Mean (SD)	55.7 (10.83)	59.0 (10.66)	57.0 (10.76)	56.7 (11.10)	55.3 (11.92)
Median (min, max)	56.0 (35, 71)	58.0 (37, 74)	57.0 (35, 74)	57.0 (24, 74)	56.0 (23, 74)
Race					
Asian	0	0	0	2 (4)	3 (5)
Native Hawaiian or Other Pacific Islander	1 (4)	0	1 (2)	1 (2)	1 (2)
White	25 (93)	16 (100)	41 (95)	48 (91)	58 (89)
Other	1 (4)	0	1 (2)	2 (4)	3 (5)
Ethnicity					
Hispanic or Latino	1 (4)	0	1 (2)	1 (2)	1 (2)
Not Hispanic or Latino	26 (96)	16 (100)	42 (98)	52 (98)	64 (98)
KPS score					
70–89	7 (26)	8 (50)	15 (35)	18 (34)	21 (32)
90–100	20 (74)	8 (50)	28 (65)	35 (66)	44 (68)
Extent of tumor resection					
Complete	10 (37)	5 (31)	15 (35)	23 (43)	29 (45)
Partial	16 (59)	9 (56)	25 (58)	27 (51)	33 (51)
Minimal	1 (4)	0	1 (2)	1 (2)	1 (2)
Unknown	0	2 (13)	2 (5)	2 (4)	2 (3)
MGMT status					
Methylated	13 (48)	7 (44)	20 (47)	23 (43)	30 (46)
Unmethylated	13 (48)	7 (44)	20 (47)	27 (51)	30 (46)
Indeterminate	0	0	0	0	1 (2)
Not reported	1 (4)	2 (13)	3 (7)	3 (6)	4 (6)

Abbreviations: BMI = body mass index; KPS = Karnofsky Performance Scale; MGMT = O6-methylguanine-DNA methyltransferase; mITT = modified intention-to-treat population; *N* = number of patients; RP2D = recommended Phase 2 dose.

Values are reported as number (%) unless otherwise indicated.

^a^Patients are summarized by doses of pexidartinib taken during combination therapy and adjuvant therapy periods. Patients reported as having discontinued the study before adjuvant treatment are summarized as having adjuvant treatment of “none.”

^b^The combined RP2D group includes all patients in Phase 1b or Phase 2 who received 800 mg, 5 days/week of pexidartinib during combination therapy. There were 10 patients from Phase 1b and 43 from Phase 2.

The median pexidartinib exposure was 102.5 days in Phase 1b and 127 days in Phase 2. In Phase 1b, the mean (SD) dose intensity of pexidartinib was slightly lower during combination therapy than during adjuvant treatment: 727.8 (95.52) mg/day versus 757.8 (200.21) mg/day. In Phase 2, the mean (SD) dose intensity of pexidartinib during combination therapy was comparable with that during adjuvant treatment: 769.5 (64.83) mg/day versus 762.1 (121.32) mg/day.

### Determination of RP2D

Of the 22 Phase 1b patients, 5 received 800 mg daily of pexidartinib (7 patients were not enrolled at this dose due to the toxicities seen in these 5 patients), 7 received 600 mg daily of pexidartinib, and 10 received 800 mg on 5 days/week of pexidartinib during the combination therapy. A total of 4 patients experienced DLTs (2 from the 800-mg daily dose cohort and 2 from the 600-mg daily dose cohort). All DLTs were hematological adverse effects, including neutropenia (including neutropenic fever), thrombocytopenia, and leukopenia. All of these events were assessed by investigators and thought to be possibly related to pexidartinib. Based on tolerability and drug exposure, the protocol oversite committee determined the RP2D to be 800 mg/day for 5 days/week during RT/TMZ (combination therapy dose), followed by 800 mg/day for 7 days/week in combination with adjuvant TMZ (adjuvant therapy dose). A total of 53 patients (10 patients in Phase 1b and 43 patients in Phase 2) received the RP2D dose of pexidartinib.

### Toxicity

AEs occurred in all 65 patients enrolled in the study (both Phase 1b and Phase 2, mITT population), with equal numbers of patients experiencing AEs attributed to pexidartinib and TMZ. Patients in both phases experienced serious AEs (SAEs): 45% in Phase 1b, 51% in Phase 2, and 49% overall. Patients in both phases were reported to have SAEs assessed by the investigators as related to pexidartinib (18% in Phase 1b, 26% in Phase 2, and 23% overall), TMZ (23% in Phase 1b, 21% in Phase 2, and 22% overall), and RT (5% in Phase 1b, 12% in Phase 2, and 9% overall). Overall, 14 (22%) patients developed AEs that led to permanent discontinuation of pexidartinib. The most frequent AE attributed to pexidartinib was fatigue (86% in Phase 1b, 49% in Phase 2, and 49% overall), followed by nausea (23% in Phase 1b, 40% in Phase 2, and 34% overall), and decreased appetite (27% in Phase 1b, 28% in Phase 2, and 28% overall) (Table 2). Two patients had AEs that resulted in death, 1 patient in Phase 1b (800 mg, 7 days/week, no adjuvant therapy dose group) with necrotizing fasciitis, and 1 in Phase 2 (800 mg, 5 days/week, 800 mg 7 days/week adjuvant therapy dose group) with a pulmonary embolism, neither of which were attributed to pexidartinib, TMZ, or RT.

**Table 2.** Number of patients experiencing adverse events related to pexidartinib (by preferred term) reported in Phase 2 (≥15% occurrence in all patients in Phase 2), the combined RP2D groups in Phase 1b and Phase 2 (mITT RP2D population), and the study overall (mITT population)

AE	Combination therapy dose/adjuvant therapy dose^b^		Phase 2 total *N* = 43	Combined RP2D^c^ Phases 1b and 2 *N* = 53	Study overall *N* = 65
	800 mg/800 mg *N* = 27	800 mg/None *N* = 16			
	5 days/7 days	5 days/none		5 days/varies	
Any event	26 (96)	14 (88)	40 (93)	50 (94)	59 (91)
Fatigue	16 (59)	5 (31)	21 (49)	28 (53)	32 (49)
Nausea	9 (33)	8 (50)	17 (40)	21 (40)	22 (34)
Decreased appetite	8 (30)	4 (25)	12 (28)	15 (28)	18 (28)
Rash	7 (26)	1 (6)	8 (19)	9 (17)	10 (15)
Neutropenia	6 (22)	1 (6)	7 (16)	9 (17)	11 (17)

Abbreviations: mITT = modified intention-to-treat population; *N* = number of patients; RP2D = recommended Phase 2 dose.

^a^Patients were counted at most 1 time per AE. AEs with relationship of “possibly related to pexidartinib” or “probably related to pexidartinib” are summarized.

^b^Patients are summarized by doses of pexidartinib taken during combination therapy and adjuvant therapy periods. Patients reported as having discontinued the study before adjuvant treatment are summarized as having adjuvant treatment of “none.”

^c^The combined RP2D group includes all patients in Phase 1b or Phase 2 who received 800 mg, 5 days/week of pexidartinib during combination therapy. There were 10 patients from Phase 1b and 43 patients from Phase 2.

A total of 60% of patients overall (64% in Phase 1b, 58% in Phase 2) experienced AEs leading to change of pexidartinib dose, with the most common in Phase 1b being thrombocytopenia and platelet count decrease (18% and 14%, respectively) and in Phase 2 being neutropenia (14%) (Table 3). Less than one-quarter of the patients (23% in Phase 1b, 21% in Phase 2, and 22% overall) experienced an AE that led to permanent discontinuation of pexidartinib.

**Table 3.** AEs leading to a change in pexidartinib (by preferred term) reported in Phase 2 (≥9% occurrence in all patients in Phase 2), the combined RP2D groups in Phase 1b and Phase 2 (mITT RP2D population), and the study overall (mITT Population)

AE^a^	Combination therapy dose/adjuvant therapy dose^b^		Phase 2 total *N* = 43	Combined RP2D^c^ Phases 1b and 2 *N* = 53 5 days/varies	Study overall *N* = 65
	800 mg/800 mg *N* = 27 5 days/7 days	800 mg/None *N* = 16 5 days/None			
Any event	16 (59)	9 (56)	25 (58)	33 (62)	39 (60)
Neutropenia	5 (19)	1 (6)	6 (14)	7 (13)	7 (11)
Alanine aminotransferase increased	1 (4)	4 (25)	5 (12)	5 (9)	6 (9)
Aspartate aminotransferase increased	0	4 (25)	4 (9)	5 (9)	6 (9)
Platelet count decreased	2 (7)	1 (6)	3 (7)	5 (9)	6 (9)
Thrombocytopenia	2 (7)	1 (6)	3 (7)	5 (9)	7 (11)

Abbreviations: mITT = modified intention-to-treat population; N = number of patients; RP2D = recommended Phase 2 dose.

^a^AEs with pexidartinib action taken of “drug temporarily withdrawn,” “drug permanently withdrawn,” or “dose reduced” are summarized.

^b^Patients are summarized by doses of pexidartinib taken during combination therapy and adjuvant therapy periods. Patients reported as having discontinued the study before adjuvant treatment are summarized as having adjuvant treatment of “none.”

^c^The combined RP2D group includes all patients in Phase 1 or Phase 2 who received 800 mg, 5 days/week of pexidartinib during combination therapy. There were 10 patients from Phase 1b and 43 patients from Phase 2.

Elevated liver enzyme changes were observed in 6 patients (9%) experiencing ALT elevations and 6 patients (9%) in AST elevations (5 patients with both AST/ALT elevations, 1 with isolated ALT, and 1 with isolated AST) that led to a change in pexidartinib dose. Of note, one patient experienced Grade 4 elevated bilirubin possibly related to pexidartinib. Clinically significant hematologic abnormalities were more commonly seen in Cycles 1, 2, and 3 than in subsequent cycles, with the most common being neutropenia (17%), thrombocytopenia (15%), and anemia (14%) in patients overall during the study. Liver function and hematological effects were observed starting in the first cycle of treatment. Most clinically significant abnormalities reported as AEs had either resolved, resolved with sequelae, or were resolving. Overall, pexidartinib was generally safe and well tolerated in patients with GBM.

### Pharmacokinetics

Nineteen (86%) Phase 1b patients had serum analysis for pexidartinib concentration. A dose-proportional increase in serum concentrations was seen for steady-state exposures from 600 mg daily to 800 mg daily. Compared with continuous dose (800 mg 7 days/week), there was a 30% lower exposure at steady state (C1D15) when pexidartinib was dosed intermittently (800 mg 5 days/week) (Supplementary Table 5, Supplementary Figure 3).

The C_max_ (ng/mL) values for all cohorts were similar when C1D8 and C1D15 were compared (Supplementary Table 5). For C1D15 levels, *C*_max_ was lowest in the 800 mg 5 days/week cohort at 4767 ng/mL, followed by the 600 mg daily dose at 5455 ng/mL, and the 800 mg daily dose at 6387 ng/mL.

### Pharmacodynamics

Serum plasma samples from 5 patients receiving pexidartinib at 800 mg daily dosing were analyzed for CSF-1 concentrations. All patient samples had an increase in plasma CSF-1 concentration after initial administration of pexidartinib (Supplementary Figure S4), with a maximum increase seen around C1D8.

Blood monocytes that are CD14^dim^CD16^+^ comprise a subset that was previously shown to be sensitive to reductions with pexidartinib treatment.^14^ The percentage of CD14^dim^CD16^+^ monocytes was analyzed in dose-comparison groups (3 patients with 600 mg/daily, 4 patients with 800 mg/daily, and 22 patients with 800 mg 5 days/week). Percentages of CD14^dim^CD16^+^ monocytes were measured in patients before pexidartinib administration and then between C1D6 and C1D8 as a follow-up (Supplementary Figure 5). Approximately half of the subjects had a decrease in CD14^dim^CD16^+^ percent of monocytes measured after initiation of pexidartinib. All 3 patients in the 600 mg 7 days/week dosing group had a decrease of CD14^dim^CD16^+^ monocytes, but such a decrease was noted in only half of the patients in the 800 mg 5 days/week dosing group. Some of these differences could be due to the timing of blood sampling relative to treatment. In the 800-mg pexidartinib for 5 days treatment arm, blood was collected 24–48 h after the last dose of pexidartinib. Only 1 of the patients at 800 mg given daily had a decrease of CD14^dim^CD16^+^ monocyte percent.

Pretreatment tumor tissue samples from a total of 44 patients were evaluated via immunohistochemistry for CSF-1 and CD163. Only 4 stained negative for CSF-1, and 25 samples had >5% CD163-positive cells.

### Efficacy

**Figure 1.**
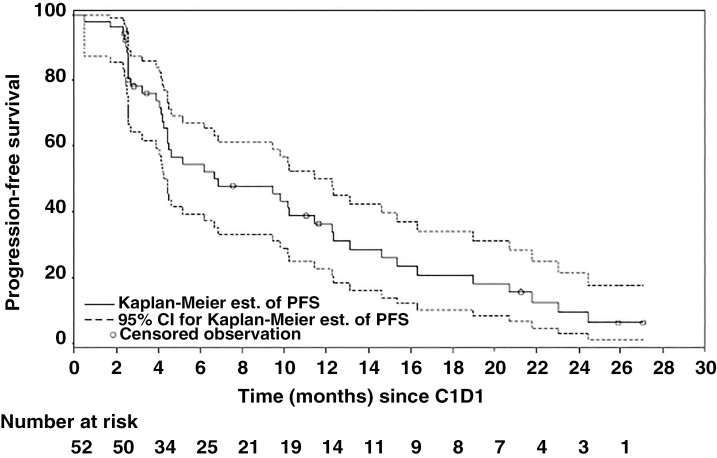
Kaplan–Meier plot of the primary endpoint, progression-free survival (PFS) (modified intention-to-treat recommended Phase 2 dose population). Subject 026_010 voluntarily withdrew after 29 days on study and did not have an MRI scan after screening, so was not evaluable for PFS. This subject is included in the number of subjects, but not in the PFS estimates. C1D1 = cycle 1 day 1.

Analysis of the primary endpoint, mPFS, was completed for the mITT RP2D population, which included all enrolled patients who received at least one dose of the RP2D of pexidartinib. The mPFS for the mITT group was 6.7 months (90% CI 4.5, 11.5; Figure 1). In the historical trials of RTOG 0525 and RTOG 0825, the median PFSs were 7.5 and 7.3 months, respectively. When comparing these historical control studies with our mITT, there were no statistically significant differences in mPFS (RTOG 0525 *P* = .456; RTOG 0825 *P* = .619).

Analysis of the secondary endpoints revealed an mOS of 13.1 months (90% CI: 11.5, 24.5) in the mITT RP2D population (Supplementary Figure 6). Using the parametric model to adjust for censoring and to directly compare OS in the current trial with those in the historical control studies, the mOS of this study was found to be 18.8 months (95% CI: 12.6, 28.0), and when compared with historical controls of RTOG 0525 (mOS 18.9 months, *P* = .389) and RTOG 0825 (mOS 16.1 months, *P* = .393), there was no statistically significant difference.

Supplementary Tables 6 and 7 show the comparisons of the estimates of mPFS and mOS with censoring for confounding therapy that were calculated with similar populations and follow-up start times as the historical controls of RTOG 0525 and RTOG 0825.

Within the unplanned subgroup analysis (Table 4), the only statistically significant difference was found when evaluating patients based on KPS (90–100 vs. 70–80). The mPFS was 11 months in patients with a KPS of 90–100 compared with 4 months in patients with a KPS of 70–89 (*P* = .003). This also held true when evaluating mOS based on KPS, with an mOS of 24 months in patients with a KPS of 90–100 and an mOS of 8 months in patients with a KPS of 70–89 (*P* < .001). Younger age appeared to influence OS positively, but the difference in mOS was not statistically significant (mOS in patients 18–64 years of 14 months vs. mOS in patients ≥65 years of 11 months; *P* = .063). Additionally, MGMT methylation subgroup analysis suggested a difference in mPFS between methylated and unmethylated groups (mPFS of 10 months vs. 4 months, respectively), but the difference was not statistically significant (*P* = .201).

**Table 4.** Summary of PFS and overall survival by subgroups (mITT RP2D population)

Subgroup	PFS				Overall survival			
	*N* ^a^	mPFS (months)	90% CI^b^	*P-*value	N^c^	mOS (months)	90% CI^b^	*P-*value
Whole	52	6.7	(4.5, 11.5)		53	13.1	(11.5, 24.5)	–
Age group								
18–64 years	39	6	(4.5, 12.4)	0.440	40	14	(12.3, NE)	0.063
65+ years	13	10	(2.7, 11.5)		13	11	(4.3, 24.5)	–
Extent of surgery								
Complete resection	23	6	(4.2, 12.3)	0.699	23	14	(9.9, 24.5)	0.969
Partial resection	26	9	(4.5, 15.4)		27	13	(7.9, NE)	–
Baseline KPS score								
70–89	18	4	(2.6, 4.5)	**0.003**	18	8	(6.9, 12.4)	**<0.001**
90–100	34	11	(6.2, 14.7)		35	24	(13.1, NE)	–
MGMT status^d^								
Methylated	23	10	(5.2, 15.4)	0.201	23	15	(9.7, NE)	0.501
Unmethylated	27	4	(3.9, 10.2)		27	12	(7.9, 20.7)	–

Abbreviations: CI = confidence interval; KPS = Karnofsky Performance Scale; MGMT = O_6_-methylguanine-DNA methyltransferase; mITT = modified intention-to-treat; mPFS = median progression-free survival; mOS = median overall survival; *N* = number of patients with data that apply or data available; NE = not estimable; RP2D = recommended Phase 2 dose.

^a^Subject 026_010 voluntarily withdrew after 29 days on study and did not have an MRI after screening, so was not evaluable for PFS.

^b^90% CI is calculated based on Kaplan–Meier methodology.

^c^Subject 020_011 continues to participate in the study but was censored on November 03, 2017.

^d^MGMT methylation status was known for 50 patients.

## Discussion

GBM is a deadly malignancy with limited therapeutic options. Many investigations have been conducted in an effort to improve survival in these patients with varying approaches including targeted agents and immunotherapies. This Phase 1b/2 study in patients with newly diagnosed GBM explored the efficacy of pexidartinib given concurrently with RT/TMZ followed in combination with adjuvant TMZ based on its potential to influence the tumor immune microenvironment and potentially serve as a radiosensitizer. Our results indicated that there was no statistically significant improvement in mPFS or mOS with the addition of pexidartinib compared with historical controls from trials RTOG 0525 and RTOG 0825.

Although pexidartinib was not efficacious against newly diagnosed GBM, our PD evaluation suggests pexidartinib can induce systemic alterations that may impact the TME. More specifically, the finding of a rise in CSF-1 levels and a decrease in CD14^dim^CD16 + monocytes after administration of pexidartinib provides evidence of the ability of pexidartinib to inhibit CSF1R systemically. Future studies will need to determine whether such systemic changes translate to meaningful alterations in the TME.

Our study was able to establish the RP2D as 800 mg on a schedule of 5 days/week during combination therapy with RT/TMZ and 800 mg on a schedule of 7 days/week during adjuvant therapy with TMZ, with liver function and hematological toxicities being most common. Most importantly, pexidartinib was found to be generally safe and well tolerated in our patient population. With the ability to achieve exposure in tumor tissue,^14^ modulate systemic alterations that may impact the TME, and have a favorable toxicity profile, pexidartinib should be considered in future trials in both newly diagnosed and recurrent GBMs, particularly as part of multitargeted therapies.

The results from the PK and PD studies suggested that more intense dosing regimens of pexidartinib correlated with an increase in drug exposure and a decrease in CD14^dim^CD16 + monocytes. More specifically, the PK studies revealed there was a 30% reduction in exposure at steady state and a decrease in AUC levels when pexidartinib was dosed intermittently (800 mg 5 days/week) compared with continuously (800 mg 7 days/week). In addition, PD evaluation suggested that there was a greater reduction in CD14^dim^CD16 + monocytes when pexidartinib was dosed continuously at 600 mg daily compared with intermittently at 800 mg 5 days/week, although these data are based on a limited number of samples in each dosing regimen. These data were not known at the time the decision was made to use the intermittent dosing regimen in the current study. However, they suggest that the 600-mg daily dosing and/or exploration of other doses and PK/PD assessments may provide additional benefit in future clinical trials.

In regards to clinical trial design, our study provides a framework for implementing a single-arm Phase 2 clinical trial. In particular, the decision in advance to use historical controls from RTOG 0525 and RTOG 0825 allowed for the planned congruence of many aspects of this clinical trial with the historical trials. More specifically, the same inclusion/exclusion criteria were applied and analysis of endpoints based on mITT was conducted as in these historical controls/trials. Furthermore, discrepancies in start times in the calculations of PFS between our trial and historical controls were accounted for to provide for an accurate comparison across the investigational and historical control arms. This approach enhanced the reliability of efficacy conclusions achieved in our study. In addition, our subgroup analysis also revealed that KPS was a significant prognostic factor whereas age and MGMT status both appeared to demonstrate some effect but were not statistically significantly different, findings that have been replicated in many other clinical trials in GBM.^23–25^ We acknowledge that the use of historical controls to assess efficacy in clinical trials is a limitation of our study based on literature in the field highlighting the changes in overall hazard profile between contemporary and historical patients.^26^

In summary, our results show that pexidartinib in combination with RT/TMZ and adjuvant TMZ lacks efficacy but is safe and well tolerated. Although a single-arm design was employed, incorporation of identical eligibility criteria and study treatment scheduling employed in defined historical control trials enhanced the reliability of our conclusions.

## Supplementary Material

vdae202_suppl_Supplementary_Tables_S1-S7_Figures_S1-S6

## Data Availability

All relevant data used to conduct this study are published in the main text or supplementary files.
