# Supplementary material for: Phase 1b/2 study of orally administered pexidartinib in combination with radiation therapy and temozolomide in patients with newly diagnosed glioblastoma
Source: Neurooncol Adv. 2024 Nov 22;6(1):vdae202. doi: 10.1093/noajnl/vdae202 (PMC11672110; doi:10.1093/noajnl/vdae202)
Supplement: vdae202_suppl_Supplementary_Tables_S1-S7_Figures_S1-S6 [file vdae202_suppl_supplementary_tables_s1-s7_figures_s1-s6.docx]

**SUPPLEMENTARY TABLES & FIGURE LEGENDS**

**SUPPLEMENTARY TABLE 1: Number and Name of Study Centers**

| **Number of Study Center** | **Name of Study Center** |
| --- | --- |
| Site 020 | Huntsman Cancer Institute, University of Utah |
| Site 021 | Northwestern University Feinberg School of Medicine |
| Site 022 | Dana Farber Cancer Institute, Center for Neuro-Oncology |
| Site 023 | Columbia University Medical Center |
| Site 024 | Henry Ford Hospital |
| Site 026 | Seattle Cancer Care Alliance |
| Site 027 | Ohio State University Wexner Medical Center |
| Site 032 | Massachusetts General Hospital (sub-site to Site 022) |
| Site 042 | Beth Israel Deaconess Medical Center (sub-site to Site 022) |

**SUPPLEMENTARY TABLE 2: Flow Cytometry Reagents to Identify CD14^dim^CD16^+^ Monocytes**

| **Fluorochrome** | **Marker** | **Manufacturer** | **Catalog Number** |
| --- | --- | --- | --- |
| Fluorescein isothiocyanate | HLA-DR | BD | 340688 |
| Phyceorythrin | CD64 | Beckman Coulter | IM3601U |
| Allophycocyanin | CD14 | BD | 340684 |
| Pacific Blue | CD16 | Invitrogen | MHCD1628 |
| Pacific Orange | CD45 | Invitrogen | MHCD4530 |

**SUPPLEMENTARY TABLE 3: Gating Procedure to Quantify CD14^dim^CD16^+^ Monocytes**

| **Step** | **Marker** | **Gate** | **Marker** | **Gate** |
| --- | --- | --- | --- | --- |
| 1 | CD45 | 500 – 50,000 | FSCA | 0 – 260,000 |
| 2 | CD14 | Ellipse | SSCA | Ellipse |
| 3 | SSCA | Auto | FSCA | Auto |
| 4 | HLA-DR | 1,000 – 200,000 | SSCA | 10,000 – 150,000 |
| 5 | CD16 | 2,000 – 50,000 | CD14 | 500 – 5,000 |

**SUPPLEMENTARY TABLE 4: Comparison of survival with historical controls**

| **Investigational Group** | **Control** |
| --- | --- |
| **mPFS** | |
| RP2D population, with PFS calculated from C1D1 (only mPFS, 95% CI, and counts of subjects and events are presented) | RP2D-0525 population, with PFS calculated from C1D1. This is compared with the Null hypothesis of 7.5 months (from RTOG 0525 study^3^) |
|  | RP2D-0525 population, with PFS calculated from RPD15. This is compared with the null hypothesis of 5.5 months (from RTOG 0525 study^3^) |
|  | RP2D-0825 population, with PFS calculated from C2D1. This is compared with the null hypothesis of 7.3 months (from RTOG 0825 study^4^) |
| **mOS** | |
| RP2D population, with OS calculated from C1D1 (only mOS, 95% CI, and counts of subjects and events are presented) | RP2D-0525 population, with OS calculated from C1D1. This was compared with the null hypothesis of 18.9 months (from RTOG 0525 study^3^) |
|  | RP2D-0525 population, with OS calculated from Rest Period Day (RPD) 15. This was compared to the null hypothesis of 16.6 months (from RTOG 0525 study^3^) |
|  | RP2D-0825 population, with OS calculated from C2D1. This was compared with the null hypothesis of 16.1 months (from RTOG 0825 study^4^) |
| **OS at 9 and 12 months** | |
| RP2D population, with OS calculated from C1D1 (only mOS, 95% CI, and counts of subjects and events are presented) | RP2D-0525 population, with OS calculated from C1D1. This was compared with an expected 13.0 deaths at 9 months and 16.4 at 12 months (based on mOS from registration of 18.9 months in the RTOG 0525 study^3^) |
|  | RP2D-0525 population, with OS calculated from RPD15. This was compared with an expected 14.3 deaths at 9 months and 18.1 deaths at 12 months (based on mOS from randomization of 16.6 months in the RTOG 0525 study^3^) |
|  | RP2D-0825 population, with OS calculated from C2D1. This was compared with an expected 14.33 deaths at 9 months and 17.8 deaths at 12 months (based on mOS from randomization of 16.1 months in the RTOG 0825 study^4^) |

**SUPPLEMENTARY TABLE 5: Mean plasma pharmacokinetic parameters of pexidartinib after oral administration for 8 days (C1D8) and 15 days (C1D15)**

| **Dose** | **Statistic** | **C1D8** | | | **C1D15** | | |
| --- | --- | --- | --- | --- | --- | --- | --- |
|  |  | **C_max_ (ng/mL)** | **AUC_0-4_ (ng•hr/mL)** | **AUC_0-5_ (ng•hr/mL)** | **C_max_ (ng/mL)** | **AUC_0-4_ (ng•hr/mL)** | **AUC_0-5_ (ng•hr/mL)** |
| 600 mg daily | N | 6 | 6 | 6 | 4 | 4 | 4 |
|  | G-Mean | 5856 | 19158 | 23959 | 5455 | 15927 | 20179 |
|  | CV% | 51.3 | 39.8 | 37.3 | 68.7 | 55.4 | 54 |
| 800 mg daily | N | 5 | 5 | 5 | 3 | 3 | 3 |
|  | G-Mean | 6473 | 20929 | 26408 | 6387 | 20074 | 24675 |
|  | CV% | 33.6 | 32.9 | 34.1 | 16.3 | 17.3 | 19.5 |
| 800 mg 5 days/week | N | 8 | 8 | 7 | 8 | 8 | 8 |
|  | G-Mean | 4374 | 13456 | 16867 | 4767 | 14065 | 17147 |
|  | CV% | 36.8 | 40.6 | 43.1 | 28.8 | 34.4 | 32.9 |

AUC = area under the curve from 0-4 hours and 0-5 hours; C_max_ = maximum observed concentration; CV% = coefficient of variance of geometric mean; G-Mean = geometric mean; N = number of patients

**SUPPLEMENTARY TABLE 6: Estimates of progression-free survival with censoring for confounding therapy**

| Analysis population | Start of follow-up | Number of subjects | Number of events^a^ | mPFS in months  (95% CI)^b^ | Null hypothesis mPFS (p-value)^c^ | HR (95% CI)^d^ |
| --- | --- | --- | --- | --- | --- | --- |
| RP2D | C1D1 | 53^e^ | 41 | 7.7 (5.6, 10.4) | -- | -- |
| RP2D-0525 | C1D1^f^ | 43 | 36 | 7.6 (5.5, 10.6) | 7.5 (0.456) | 0.98 (0.71, 1.36) |
| RP2D-0825 | C2D1 | 49 | 40 | 7.0 (5.1, 9.5) | 7.3 (0.619) | 1.05 (0.77, 1.43) |
| RP2D-0525 | RPD15 | 43 | 36 | 6.1 (4.4, 8.5) | 5.5 (0.272) | 0.90 (0.65, 1.26) |

^a^Disease progression or death.

^b^Estimates of mPFS (months) based on the fit of an intercept-only exponential regression survival model.

^c^Alternative hypothesis for this current study is that mPFS for these subjects exceeds the indicatedNull value for mPFS of the RTOG 0525 and RTOG 0825 studies. The p-value is one-sided, based on the one-sample log-rank test outlined in Finkelstein et al.,^1^ assuming an intercept-only exponential regression survival model under theNull hypothesis.

^d^HR is the estimated hazard rate for these subjects relative to the hazard rate corresponding with the indicatedNull hypothesis for mPFS, assuming an intercept-only exponential regression survival model.

^e^Subject 026_010 voluntarily withdrew after 29 days on study and did not have an MRI after screening, so was not evaluable for PFS. This subject is included in the number of subjects, but not in the PFS estimates.

^f^Among subjects with follow-up extending beyond RPD15.

Abbreviations: mPFS = median progression-free survival; CI = confidence interval; HR = hazard ratio; C1D1 = Cycle 1, Day 1; C2D1 = Cycle 2, Day 1; RPD15 = Rest period, Day 15; RP2D = recommended Phase 2 dose; RP2D-0525 = recommended Phase 2 dose in the RTOG 0525 study; RP2D-0825 = recommended Phase 2 dose in the RTOG 0825 study; RTOG = Radiation Therapy Oncology Group

**SUPPLEMENTARY TABLE 7: Estimates of overall survival with censoring for confounding therapy**

| Analysis population | Start of follow-up | Number of subjects | Number of deaths | mOS in months (95% CI)^a^ | Null hypothesis mPFS (p-value)^b^ | HR (95% CI)^c^ |
| --- | --- | --- | --- | --- | --- | --- |
| RP2D | C1D1 | 53 | 24 | 18.8 (12.6, 28.0) | -- | -- |
| RP2D-0525 | C1D1^d^ | 43 | 19 | 20.2 (12.9, 31.6) | 18.9 (0.389) | 0.94 (0.60, 1.47) |
| RP2D-0825 | C2D1 | 49 | 23 | 17.0 (11.3, 25.6) | 16.1 (0.393) | 0.94 (0.63, 1.42) |
| RP2D-0525 | RPD15 | 43 | 19 | 16.9 (10.8, 26.5) | 16.6 (0.469) | 0.98 (0.63, 1.54) |

^a^Estimates of mOS (months) based on the fit of an intercept-only exponential regression survival model.

^b^Alternative hypothesis for this current study is that mOS for these subjects exceeds the indicatedNull value for mOS of the RTOG 0525 and RTOG 0825 studies. The p-value is one-sided, based on the one-sample log-rank test outlined in Finkelstein et al.,^1^ assuming an intercept-only exponential regression survival model under theNull hypothesis.

^c^HR is the estimated hazard rate for these subjects relative to the hazard rate corresponding with the indicatedNull hypothesis for mOS, assuming an intercept-only exponential regression survival model.

^d^Among subjects with follow-up extending beyond RPD15.

Abbreviations: mOS = median overall survival; mPFS = median progression-free survival; CI = confidence interval; HR = hazard ratio; C1D1 = Cycle 1, Day 1; C2D1 = Cycle 2, Day 1; RPD15 = Rest period, Day 15; RP2D = recommended Phase 2 dose; RP2D-0525 = recommended Phase 2 dose in the RTOG 0525 study; RP2D-0825 = recommended Phase 2 dose in the RTOG 0825 study; = Rest period, Day 15; RTOG = Radiation Therapy Oncology Group

**References**

1. Finkelstein DM, Muzikansky A, Schoenfeld DA. Comparing survival of a sample to that of a standard population. *J Natl Cancer Inst* 2003;95(19):1434-1439.

**SUPPLEMENTARY FIGURE LEGEND**

**SUPPLEMENTARY FIGURE 1: Phase 2 treatment regimens for pexidartinib (PLX3397), radiotherapy (RT), and temozolomide (TMZ).** Abbreviations: C1D1 = Cycle 1 day 1; RP2D = recommended phase 2 dose; M-F = 5 days/week; QD = once daily; BID = twice daily.

**SUPPLEMENTARY FIGURE 2: Enrollment and patient disposition.** Patients are summarized by doses of pexidartinib taken during combination therapy/adjuvant therapy periods. Patients reported as having discontinued the study before adjuvant treatment are summarized as having adjuvant treatment of “none.” The combined recommended phase 2 dose group includes all patients in Phase 1 or Phase 2 who received 800 mg × 5d pexidartinib during combination therapy. Abbreviations: AE = adverse event; d = day; dis.prog. = disease progression; discontin. = discontinued; inv. dec.: Investigator decision; N = number of patients; non-comp. = non-compliance; RP2D = recommended Phase 2 dose; sub. dec. = subject decision; vol. withdr. = voluntary withdrawal.

**SUPPLEMENTARY FIGURE 3: Mean plasma concentrations of pexidartinib after oral administration for 8 days (C1D8) and 15 days (C1D15).**

**SUPPLEMENTARY FIGURE 4: Plasma CSF-1 concentrations**. Plasma samples from the 5 patients who received 800 mg pexidartinib daily were analyzed. Every patient showed an increase in plasma CSF-1 levels after one week of pexidartinib treatment. (A) CSF-1 levels of individual patients. (B) Boxplot showing the distribution of CSF-1 levels before (C1D1) and after (C1D8 and C2D1) pexidartinib treatment.

**SUPPLEMENTARY FIGURE 5: CD14^dim^CD16^+^ (% of monocytes)**. Circulating CD14^dim^CD16^+^ monocytes were analyzed for 3 patients who received 600 mg pexidartinib daily, 4 patients who received 800 mg pexidartinib daily, and 22 patients who received 800 mg pexidartinib 5 days/week (the RP2D dose). (A) CD14^dim^CD16^+^ levels for individual patients. (B) Boxplot showing the distribution. (C) After approximately 1 week of pexidartinib, nine subjects showed a decrease in circulating CD14^dim^CD16^+^ monocytes of ~5% or more and only 3 subjects showed an increase of 5% or more. Of the 3 subjects with increased CD14^dim^CD16^+^ monocytes, 2 were in the treatment arm that received pexidartinib for 5 days and had therefore been off of pexidartinib treatment for approximately 24–48 hours prior to CD14^dim^CD16^+^ blood sampling.

**SUPPLEMENTARY FIGURE 6: Secondary endpoint, Kaplan-Meier plot of overall survival (modified intention-to-treat, recommended Phase 2 dose population)**
